# A randomized trial of human C1 inhibitor prophylaxis in children with hereditary angioedema

**DOI:** 10.1111/pai.13060

**Published:** 2019-05-29

**Authors:** Emel Aygören‐Pürsün, Daniel F. Soteres, Sandra A. Nieto‐Martinez, Jim Christensen, Kraig W. Jacobson, Dumitru Moldovan, Arthur Van Leerberghe, Yongqiang Tang, Peng Lu, Moshe Vardi, Jennifer Schranz, Inmaculada Martinez‐Saguer

**Affiliations:** ^1^ Department for Children and Adolescents, Angioedema Centre, University Hospital Frankfurt Goethe University Frankfurt Germany; ^2^ Asthma and Allergy Associates P.C. Colorado Springs Colorado; ^3^ Genetics Unit of Nutrition National Institute of Pediatrics Mexico City Mexico; ^4^ Nevada Access to Research & Education Society Las Vegas Nevada; ^5^ Oregon Allergy Associates Allergy and Asthma Research Group Eugene Oregon; ^6^ Mures County Hospital University of Medicine and Pharmacy of Târgu Mureș Târgu Mureș Romania; ^7^ Shire, a Takeda Company Lexington Massachusetts; ^8^ HRZM Hemophilia Center Rhein Main Mörfelden‐Walldorf Germany

**Keywords:** C1 esterase inhibitor (human), efficacy, health‐related quality of life, hereditary angioedema, pediatric patients, phase 3 study, prophylaxis, safety

## Abstract

**Background:**

Patients with hereditary angioedema with C1 inhibitor deficiency or dysfunction have burdensome recurrent angioedema attacks. The safety, efficacy, and health‐related quality of life (HRQoL) outcomes of C1 inhibitor (C1‐INH) prophylaxis (intravenously administered) in patients aged 6‐11 years were investigated.

**Methods:**

Eligible patients were enrolled in a randomized, single‐blind, crossover, phase 3 trial. After a 12‐week baseline observation period (BOP), patients received 500 or 1000 U C1‐INH, twice weekly, for 12 weeks before crossing over to the alternate dose for 12 weeks. The primary efficacy end‐point was the monthly normalized number of angioedema attacks (NNA). HRQoL was assessed using the EuroQoL 5‐dimensional descriptive system youth version and visual analog scale (EQ‐VAS).

**Results:**

Twelve randomized patients had a median (range) age of 10.0 (7‐11) years. Mean (SD) percentage reduction in monthly NNA from BOP was 71.1% (27.1%) with 500 U and 84.5% (20.0%) with 1000 U C1‐INH. Mean (SD) within‐patient difference (−0.4 [0.58]) for monthly NNA with both doses was significant (*P *= 0.035 [90% CI, −0.706 to −0.102]). Cumulative attack severity, cumulative daily severity, and number of acute attacks treated were reduced. No serious adverse events or discontinuations occurred. Mean EQ‐VAS change from BOP to week 9 of treatment (500 U C1‐INH, 10.4; 1000 U C1‐INH, 21.6) was greater than the minimal important difference, indicating a meaningful HRQoL change.

**Conclusions:**

C1‐INH prophylaxis was effective, safe, and well tolerated in children aged 6‐11 years experiencing recurrent angioedema attacks. A post hoc analysis indicated a meaningful improvement in HRQoL with C1‐INH.

**Trial registration:**

ClinicalTrials.gov identifier NCT02052141.

**Key Message**

Before this study was initiated, intravenous C1‐INH (Shire) was approved for routine prophylaxis against HAE attacks in the United States and in the European Union in adolescent and adult patients, but not in pediatric patients <12 years of age. This study shows that patients aged 6‐11 years with recurrent and burdensome hereditary angioedema attacks would benefit from prophylaxis with twice‐weekly C1‐INH (500 or 1000 U) as it is efficacious and safe, and may lead to a meaningful improvement in health‐related quality of life.

## 1 INTRODUCTION

Hereditary angioedema with C1 inhibitor deficiency or dysfunction (C1‐INH‐HAE) is a rare inherited disease with an estimated prevalence of 1:50 000,^1^ characterized by episodic swelling of the skin, abdomen, and larynx.^2^, ^3^, ^4^, ^5^ C1‐INH‐HAE's clinical manifestations including age of first symptoms, and attack location, frequency, and severity, are heterogeneous.^6^, ^7^, ^8^ Approximately 50% of patients have potentially fatal laryngeal attacks.^9^, ^10^ In a European survey of patients aged ≥12 years, recurrent acute angioedema attacks impaired health‐related quality of life (HRQoL) to a similar extent to other serious chronic diseases.^11^ The HRQoL of patients aged 3‐18 years can be impaired relative to healthy controls, especially in school and physical domains, due to the frequency and site of angioedema attacks.^12^ Questionnaires completed by/for patients aged 5‐18 years in Israel and Hungary found that children and adolescents had higher anxiety traits correlating with HRQoL impairment.^13^

International World Allergy Organization/European Academy of Allergy and Clinical Immunology guidelines for managing C1‐INH‐HAE recommend C1‐INH for first‐line long‐term prophylaxis.^14^ Although pediatric patients were included in prior studies of C1‐INH,^15^ no dedicated randomized clinical trials specifically assessed children aged ≥6 to <12 years. As C1‐INH was safely used in children in uncontrolled studies or studies non‐specific to a pediatric cohort, C1‐INH is recommended for emergencies.^16^

Recent consensus guidelines indicated the need for phase 3 clinical trials specifically targeting pediatric populations.^17^ In Europe and the United States, C1‐INH (Cinryze; Shire) was recently approved for routine prophylaxis in patients aged ≥6 years, but before this study was initiated, it was only approved for adult and adolescent patients. Interim results of this study were previously published.^18^ This article describes full study results in 12 patients.

## 2 METHODS

The randomized, phase 3, single‐blind, crossover study involved 10 sites in the United States, European Union, Mexico, and Israel (NCT02052141). Patient assent and written informed consent from parents/legal guardians were obtained, and the Institutional Review Board approved study materials. The study adhered to the International Conference on Harmonisation Good Clinical Practice guidelines, the principles of the Declaration of Helsinki, and other local ethical and legal requirements.

Eligible patients were aged ≥6 to <12 years with a confirmed HAE type I/II diagnosis, functional C1‐INH level <50% of normal, and an average of ≥1.0 (≥2.0 in Germany) attacks/month of moderate or severe intensity or requiring acute treatment. A randomization patient number of 12 was targeted due to the ability to enroll patients aged 6‐11 years with C1‐INH‐HAE.

A 12‐week baseline observation period (BOP) occurred after screening. Using the sponsor's randomization schedule, patients were assigned with equal probability to 500 or 1000 U C1‐INH (intravenously administered by qualified personnel at the investigational site or at the patient's home/other location), every 3‐4 days for 12 weeks before switching to the alternate dose for 12 weeks with no washout between the doses. Only patients and parents/caregivers were blinded to treatment sequence and dose. Attacks were classified as mild, moderate, or severe (severity score 1, 2, or 3). Adverse events were recorded throughout the study.

Efficacy analyses included patients in the safety set with ≥1 post‐baseline primary efficacy assessment. The primary efficacy end‐point was the monthly normalized number of attacks (NNA) in a 12‐week treatment period. Secondary efficacy end‐points were cumulative attack severity (sum of the maximum symptom severity score recorded for each attack), cumulative daily severity (sum of the severity scores recorded for each day of symptoms), and number of attacks receiving acute treatment. Primary and secondary end‐points were normalized for number of days a patient participated in a given period, expressed as a monthly frequency. C1‐INH doses were compared using a 2‐sided paired *t* test conducted at *α* = 0.1. A post hoc analysis of the patient proportion with a ≥50%, ≥70%, and ≥90% reduction in NNA relative to baseline was performed.

To assess a potential carryover effect, primary and secondary efficacy end‐points excluding the first 2 weeks of the second treatment period and then the first 2 weeks of both periods were analyzed. Treatment and sequence effects for all end‐points (without removing the first 2 weeks of the second treatment period) also were tested using a mixed‐effects model at *α* = 0.1, with treatment, sequence, and their interactions as fixed effects and assessments for same patient within sequence as repeated measurements.

HRQoL was assessed using the EuroQol 5‐dimensional descriptive system youth version (EQ‐5D‐Y) at screening, weeks 5 and 9 of the BOP, weeks 1, 5, and 9 of both treatment periods, and each day of an angioedema attack. Patients/caregivers used electronic study diaries to record information on angioedema attacks and complete EQ‐5D‐Y questionnaires. The EQ‐5D‐Y is a descriptive system comprising five dimensions in child‐friendly language (“mobility,” “looking after myself,” “doing usual activities,” “having pain/discomfort,” and “feeling worried, sad, or unhappy”). Each dimension has three problem severity levels (“no problems,” “some problems,” and “a lot of problems”). The EQ‐5D‐Y includes a visual analog scale (EQ‐VAS) of overall health ranging from 0 (worst imaginable health) to 100 (best imaginable health). EQ‐5D‐Y responses and EQ‐VAS scores were summarized by combining those for the same dose for both periods and scheduled visit using descriptive statistics. EQ‐VAS score differences from the BOP (average of all pre‐dose visits during the BOP) to week 9 of each treatment period were summarized. The minimal important difference (MID) was estimated using half the SD of the EQ‐VAS value during the BOP.^19^, ^20^

## 3 RESULTS

### 3.1 Patient demographics and baseline characteristics

Between March 2014 and May 2017, 4 of 16 screened patients failed to meet the randomization criteria and 12 were randomized and completed the study. Table 1 summarizes patient demographics and baseline characteristics. Most patients received on‐demand acute treatment for an angioedema attack within 3 months pre‐screening. No patients received long‐term prophylaxis for HAE with 3 months before screening or the BOP.

**Table 1:** Patient demographics, baseline characteristics, and characteristics of attacks that occurred up to 3 mo before patient screening

Characteristic		Patients n = 12
Age, y, median (range)		10.0 (7‐11)
Sex, n (%)	Female	7 (58.3)
Race, n (%)	White	11 (91.7)
	Mixed: Black, White	1 (8.3)
Body mass index, kg/m^2^, median (range)		18.6 (13.1‐28.2)
HAE type I, n (%)		12 (100)
Patients with first‐degree relative with HAE,^a^ n (%)		9 (75.0)
Patients who received HAE therapy 9 mo before screening, n (%)		8 (66.7)
Attacks that occurred in 3 mo before screening		
Number of attacks, median (range)		5.5 (3‐48)
Locations affected by attacks, n (%)	Upper airway	3 (25.0)
	Gastrointestinal tract or abdomen	12 (100)
	Genitourinary	2 (16.7)
	Facial	7 (58.3)
	Extremity or peripheral	10 (83.3)
Average severity of attacks experienced by patient, n (%)	Moderate	9 (75.0)
	Severe	3 (25.0)
Average duration of attack, days, median (range)		1.5 (1‐3)
Patients needing acute treatment for HAE attack, n (%)		11 (91.7)

HAE, hereditary angioedema.

Six patients had ≥1 sibling diagnosed with HAE.

a Eight patients had a mother, and 1 had a father, diagnosed with HAE.

#### 3.1.1 Greater reduction in monthly NNA with 1000 vs 500 U C1‐INH relative to baseline

**Figure 1:**
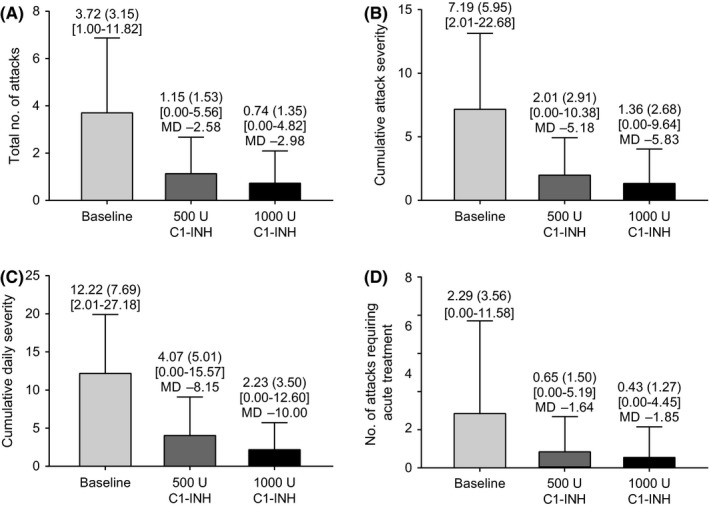
A, Total number of HAE attacks. B, Cumulative attack severity. C, Cumulative daily severity. D, Number of attacks needing acute treatment. Data (n = 12) normalized per month. Mean (SD) values shown at top of each bar. Minimum/maximum values shown in square brackets. Mean difference (MD) between baseline and treatment period shown

Five patients were randomized to the 500/1000 U C1‐INH treatment sequence and 7 to the 1000/500 U sequence. Patients had a mean (SD) NNA of 3.72 (3.15) during the BOP (Figure 1A). The median percentage reduction in NNA from baseline was 76.2% with 500 U C1‐INH and 87.4% with 1000 U. The reduction in NNA was significantly greater (*P* = 0.035 [90% CI, −0.706 to −0.102]) with 1000 vs 500 U C1‐INH (mean [SD] within‐patient difference, −0.4 [0.58]).

**Figure 2:**
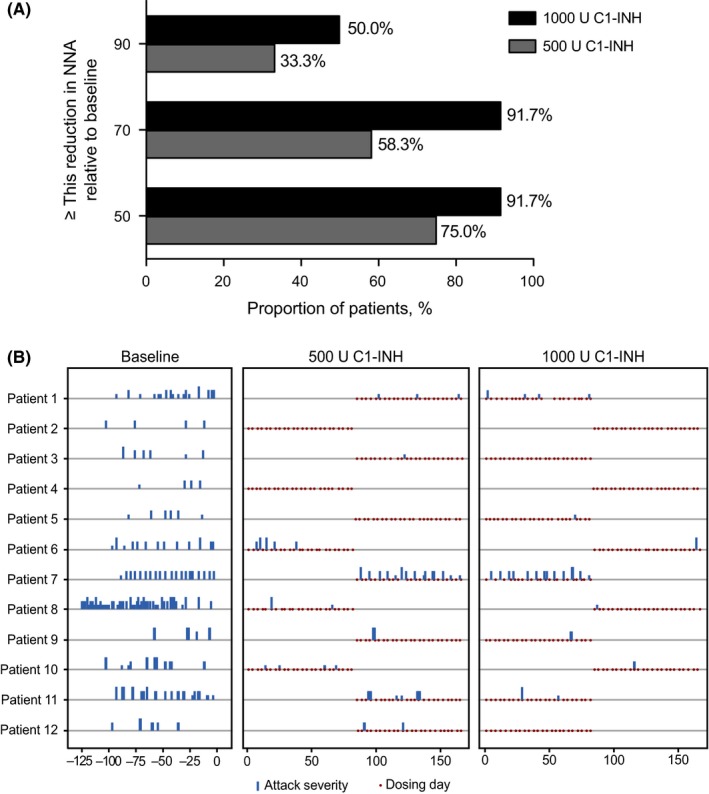
A, Clinical response rate. Proportion of patients achieving at least 50%, 70%, or 90% reduction relative to baseline in NNA. B, Angioedema attack and severity profile of patients during baseline observation period and during treatment with 500 and 1000 U C1‐INH. Data for full analysis set shown (n* = *12)

A ≥70% reduction in NNA from baseline was achieved by 58.3% of patients with 500 U C1‐INH and 91.7% of patients with 1000 U (Figure 2A).

#### 3.1.2 Patients with no attacks

**Figure 3:**
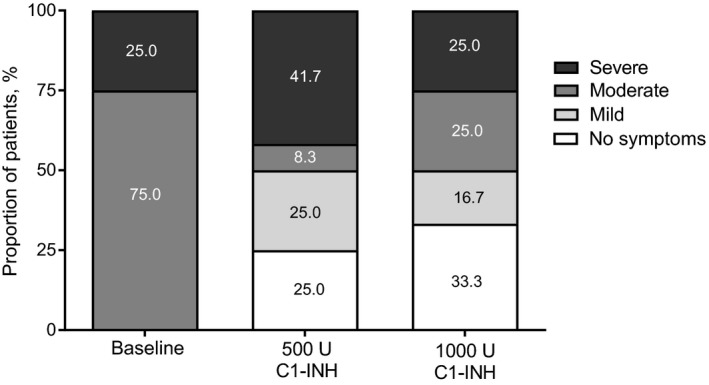
Proportion of patients by attack severity during baseline observation period and during treatment with 500 and 1000 U C1‐INH. Data for full analysis set shown (n = 12)

Five of 12 patients (42%) had no attacks in ≥1 treatment period with either dose (Figure 2B). Three of 12 patients (25%; patients 2, 4, and 5) had no attacks with 500 U C1‐INH and 4/12 patients (33%; patients 2, 3, 4, and 12) with 1000 U (Figure 3). The mean (SD) normalized number of attack‐free days was 28.2 (2.7) with 500 U C1‐INH and 29.3 (1.7) with 1000 U.

#### 3.1.3 Attack severity and number of acute attacks treated with rescue medication were reduced

Monthly normalized cumulative attack severity, cumulative daily severity, and number of treated acute attacks were reduced with both doses compared with the BOP (Figure 1B‐D). Mean (SD) change from the BOP in cumulative attack severity was −5.2 (5.4) [90% CI, −8.0 to −2.4] with 500 U C1‐INH and −5.8 (5.5) [90% CI, −8.7 to −3.0] with 1000 U. Mean (SD) change from the BOP in cumulative daily severity was −8.2 (6.5) [90% CI, −11.5 to −4.8] with 500 U C1‐INH and −10.0 (7.5) [90% CI, −13.9 to −6.1] with 1000 U. Mean (SD) change from the BOP in number of treated acute attacks was −1.6 (3.0) [90% CI, −3.2 to −0.1] with 500 U C1‐INH and −1.9 (3.1) [90% CI, −3.5 to −0.2] with 1000 U.

Using a 2‐sided paired *t* test at *α* = 0.1, there was a significant difference between either dose for number of angioedema attacks (*P* = 0.03), cumulative attack severity (*P* = 0.05), cumulative daily severity (*P* = 0.04), and number of treated acute attacks (*P* = 0.07). These results were consistent when analyzed using a mixed‐effects model (Appendix 1), and no significant differences for sequence effect were observed. Two sensitivity analyses conducted to assess any carryover effect, one excluding the first 2 weeks of the second treatment period and the second without the first 2 weeks of both treatment periods, showed a significant difference (using a mixed‐effects model at *α* = 0.1) between either dose for number of angioedema attacks (*P* = 0.03), cumulative attack severity (*P* = 0.07), and number of treated acute attacks (*P* = 0.05), and no significant differences for sequence effect. There was no significant difference between the doses for cumulative daily severity.

#### 3.1.4 Both C1‐INH doses were safe and well tolerated

Breakthrough HAE attacks, captured as treatment‐emergent adverse events (TEAEs), occurred in 10/12 (83.3%) patients. Within 24 hours of administration, the most common non–HAE attack TEAEs were fatigue and/or irritability (Table 2). One HAE attack in one patient was considered by the investigator as related to C1‐INH (1000 U), and in two patients, mainly mild TEAEs of fatigue (18 events) and irritability (14 events) were considered related to both C1‐INH doses. Eleven severe breakthrough HAE attacks occurred in five patients (six during treatment with 500 U C1‐INH, three with 1000 U, and two during follow‐up). Furthermore, a severe TEAE (dental caries) occurred in one patient.

**Table 2:** Treatment‐emergent adverse events

	Patients who experienced ≥1 event of that type, n (%) n = 12

Any type of TEAE (within 24 h of administration)	11 (91.7)
HAE attack TEAE (within 24 h of administration)	4 (33.3)
Non–HAE attack TEAE (within 24 h of administration)	
Fatigue	2 (16.7)
Irritability	2 (16.7)
Diarrhea	1 (8.3)
Gingivitis	1 (8.3)
Tonsillitis	1 (8.3)
Infusion‐site pain	1 (8.3)
Excoriation	1 (8.3)
Post‐traumatic neck syndrome	1 (8.3)
Decreased appetite	1 (8.3)
Epistaxis	1 (8.3)
Oropharyngeal pain	1 (8.3)
Erythema	1 (8.3)
Coccydynia	1 (8.3)
Vascular pain	1 (8.3)
Any type of TEAE related to study drug	4 (33.3)
HAE attack TEAE related to study drug	1 (8.3)
Non–HAE attack TEAE related to study drug	
Fatigue	2 (16.7)
Irritability	2 (16.7)
Diarrhea	1 (8.3)
Erythema	1 (8.3)
Pruritus	1 (8.3)
Any type of TEAE by maximum severity	
Mild^a^	1 (8.3)
Moderate^b^	4 (33.3)
Severe^c^, ^d^	6 (50.0)
HAE attack TEAE by maximum severity	
Mild^a^	2 (16.7)
Moderate^b^	3 (25.0)
Severe^c^, ^d^	5 (41.7)
TEAE during study drug administration	0
Any serious TEAE	0
TEAE leading to study drug discontinuation	0

HAE, hereditary angioedema; TEAE, treatment‐emergent adverse event.

a Mild attacks defined as those with noticeable signs and symptoms of an angioedema attack easily tolerated without interfering with routine activities.

b Moderate attacks defined as those that interfered with the patient's ability to attend school or participate in family life and social or recreational activities.

c Severe attacks defined as those that significantly limited the patient's ability to attend school or participate in family life and social or recreational activities.

d Six patients had 12 severe TEAEs: 11 HAE attacks (6 during treatment with 500 U C1‐INH and 2 during follow‐up, and 3 during treatment with 1000 U C1‐INH) and 1 report of dental caries. Patients were counted by the treatment most recently taken when the event occurred. Patients were counted once per category per treatment. TEAEs were defined as events with a start date and time on or after the first dose of C1‐INH and up to 7 d after the last dose of C1‐INH, or with an increase in severity on or after the date and time of first dose.

No serious TEAEs or discontinuations occurred (Table 2). All patients tested negative for anti–C1‐INH antibodies following 6 months of total exposure to study drug (2.7 person‐years), and no thrombotic or thromboembolic TEAEs occurred.

#### 3.1.5 Patients' HRQoL improved, particularly with 1000 U C1‐INH

**Figure 4:**
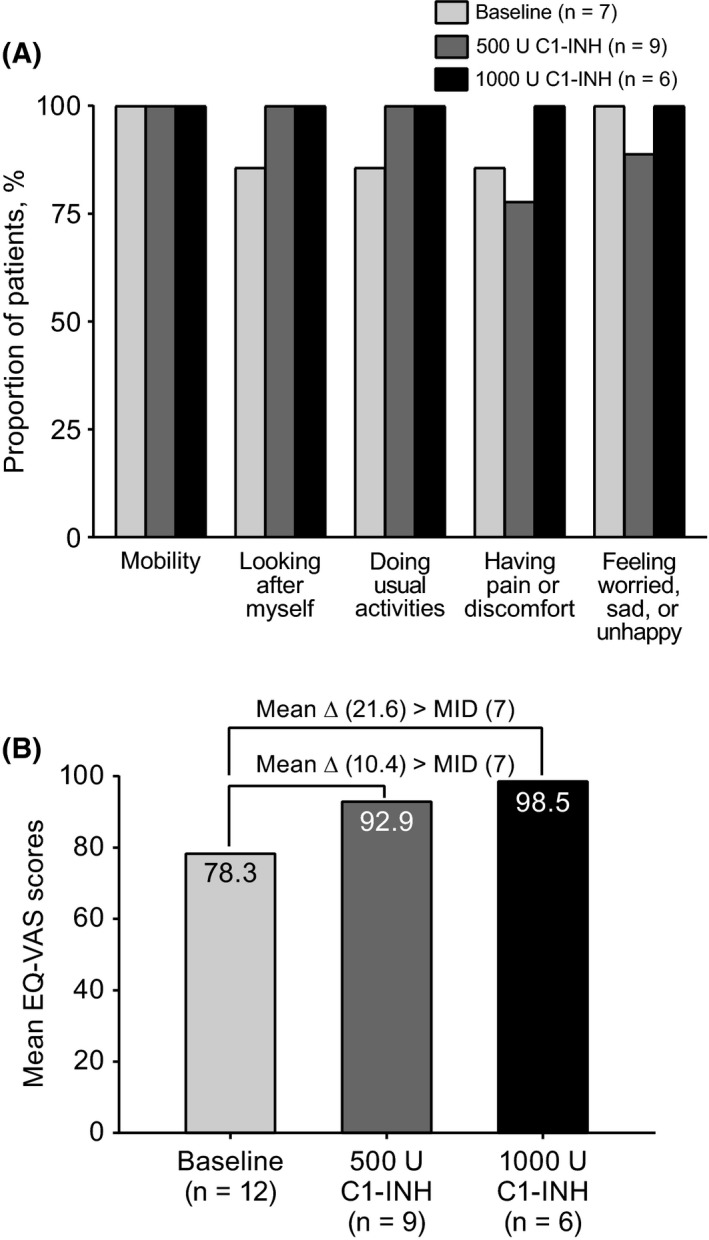
A, Proportion of patients who responded “no problems” to EQ‐5D‐Y questionnaire at scheduled week 9 visit. B, Mean EQ‐VAS scores at baseline and week 9 of treatment. Data shown for patients in full analysis set with valid non‐missing result at time point and dimension. EQ‐VAS score change ≥7 considered MID. Δ: change

Most patients experienced no problems in any EQ‐5D‐Y dimension. At screening and weeks 5 and 9 of the BOP, ≤10.0% of patients reported problems with mobility and/or feeling worried, sad, or unhappy; ≤14.3% reported problems with self‐care and doing usual activities; and ≤33.3% reported problems with pain/discomfort. During treatment with 500 U C1‐INH (weeks 5 and 9), no patients reported problems with mobility, self‐care, and doing usual activities; 11.1% reported feeling worried, sad, or unhappy; and ≤22.2% reported having pain or discomfort. During treatment with 1000 U C1‐INH (weeks 5 and 9), no patients reported problems with any of the five dimensions. For simplicity, the patient proportion with no problems at week 9 is shown in Figure 4A.

The MID for the EQ‐VAS was calculated as half the SD at baseline (13.8); therefore, a change in EQ‐VAS score >7 was considered the minimal important change, with higher EQ‐VAS scores indicating better HRQoL. At week 9, the mean (SD) change in EQ‐VAS score from baseline with 500 and 1000 U C1‐INH was greater than the MID indicating a meaningful change (ie, 10.4 [19.0] with 500 U C1‐INH and 21.6 [13.4] with 1000 U; Figure 4B).

## 4 DISCUSSION

World Allergy Organization consensus guidelines highlighted the optimization of existing long‐term prophylactic and on‐demand therapies, for example, by dose‐ranging and pediatric‐specific studies, an unmet need of HAE management.^14^ In a post hoc analysis of two placebo‐controlled and two open‐label extension studies of 1000 U C1‐INH, the mean number of attacks in four patients (aged 9‐17 years) was 13.0 with placebo and 7.0 with C1‐INH over 12 weeks, and the median number of monthly attacks in 23 patients (aged 2‐17 years) was 3.0 pre‐enrollment and 0.39 post‐treatment.^15^ In the open‐label study, 87% of patients aged 2‐17 years experienced <1 monthly attack and 22% had no attacks.^15^ The reduction in the number and frequency of attacks with twice‐weekly C1‐INH is expected to improve the HRQoL of patients with C1‐INH‐HAE.^12^

In this first blinded, randomized, controlled trial of C1‐INH for prophylaxis targeting patients aged 6‐11 years, the interim analysis in six patients showed that both doses reduced the monthly number of attacks by a mean of 1.89 compared with the BOP.^18^ In the complete study described here, mean (percentage) reduction in monthly number of attacks relative to the BOP was 2.58 (71.1%) with 500 U C1‐INH and 2.98 (84.5%) with 1000 U C1‐INH. In both analyses (interim and complete), angioedema attacks were generally less severe with prophylaxis and fewer attacks required rescue medication. As in the interim analysis, both C1‐INH doses were efficacious, safe, and well tolerated in children with C1‐INH‐HAE, but the complete study demonstrated that the 1000 U dose is more efficacious. Although some patients had no attacks with 500 U C1‐INH, 1000 U C1‐INH induced a statistically significant effect in reducing the number and severity of attacks and number of treated acute attacks. Dose and frequency adjustments prevent breakthrough attacks in adults effectively.^21^

No attacks occurred in this study for 25% of patients with 500 U C1‐INH and 33% with 1000 U. Similarly, in a placebo‐controlled phase 3 trial of twice‐weekly 1000 U C1‐INH, 18% of 22 patients had no attacks,^21^, ^22^ and in an open‐label extension study, 35% of 146 patients had no attacks.^23^

The study's safety findings are generally consistent with those in the interim analysis and with pediatric and adult subgroups in other C1‐INH studies.^18^, ^24^

In a recent study, 33 children with C1‐INH‐HAE had a significantly higher anxiety state and trait than 52 healthy controls, which inversely correlated with HRQoL.^13^ Few studies have assessed prophylaxis’ effect on the HRQoL of patients with C1‐INH‐HAE. Lumry et al used the Short Form 36 (SF‐36) version 1.0 questionnaire to evaluate HRQoL in 22 patients at the start and end of two successive 12‐week periods, where patients received placebo or twice‐weekly 1000 U C1‐INH. For 16 patients, least‐square mean differences between treatment and placebo in norm‐based SF‐36 scores at the end of each treatment period were 6.55 (*P *= 0.015) for physical component summary score and 8.70 (*P *= 0.019) for mental component summary score, indicating that twice‐weekly C1‐INH improved HRQoL.^25^

To our knowledge, our study is the first phase 3 clinical trial evaluating HRQoL in patients aged 6‐11 years receiving C1‐INH to prevent angioedema attacks. Patients receiving 1000 U C1‐INH reported no problems in all 5 EQ‐5D domains, and all patients receiving 500 U had no problems with mobility, self‐care, and doing usual activities at weeks 5 and 9 of treatment. Our HRQoL assessments and those by Lumry et al are limited, as SF‐36 and EQ‐5D‐Y are not angioedema‐specific HRQoL assessment tools that fully address characteristic burdens of C1‐INH‐HAE. Adapting the EQ‐5D questionnaire to assess HRQoL in adults with HAE was previously investigated^11^; however, estimates for children are unavailable. Our analysis also did not test a specific hypothesis for HRQoL and used descriptive statistics. At week 9 of treatment, EQ‐VAS data were missing for 25% of patients receiving 500 U C1‐INH and 50% of patients receiving 1000 U C1‐INH. Due to the disease's rarity and the patient age targeted, the sample size was small, limiting the study.

Overall, prophylaxis with 1000 U C1‐INH was statistically superior to 500 U C1‐INH in reducing monthly NNA and provided better outcomes, but some patients also had excellent results with the lower dose. Both doses were effective, safe, and well tolerated and reduced the burden of disease for patients aged 6‐11 years experiencing recurrent angioedema attacks.

## CONFLICT OF INTEREST

Emel Aygören‐Pürsün has received honoraria, research funding, and/or travel grants from, and/or served as a consultant for, Adverum, BioCryst, CSL Behring, Pharming Technologies, KalVista Pharmaceuticals, and Shire. Daniel F. Soteres is a speaker and has participated in advisory boards for Shire. Sandra A. Nieto‐Martinez is a speaker for, has received honoraria from, and has participated in advisory boards for Shire, and has received travel grants from CSL Behring, Pharming Technologies, and Shire. Kraig W. Jacobson has participated in clinical trials for Shire. Dumitru Moldovan received research funding and travel grants from CSL Behring, Pharming Technologies, and Shire HGT and unrestricted educational grants from CSL Behring, Pharming Technologies, Shire HGT, and Swedish Orphan Biovitrum and served as a consultant for Pharming Technologies and Swedish Orphan Biovitrum. Inmaculada Martinez‐Saguer has received honoraria, research funding, and travel grants from BioCryst, CSL Behring, Pharming Technologies, and Shire and/or served as a consultant for these companies. Arthur Van Leerberghe, Yongqiang Tang, Peng Lu, and Moshe Vardi are full‐time employees of Shire, a Takeda company (Lexington, MA, USA). Jennifer Schranz was a full‐time employee of Shire, a Takeda company (Lexington, MA, USA), at the time of this study. Jim Christensen has indicated that he has no potential conflicts of interest to disclose.

## AUTHOR CONTRIBUTIONS

Dr Aygören‐Pürsün conceptualized and designed the study, collected data, and critically reviewed and revised the manuscript for important intellectual content. Dr Soteres, Dr Nieto‐Martinez, Dr Christensen, Dr Jacobson, Dr Moldovan, and Dr Martinez‐Saguer collected data and critically reviewed and revised the manuscript for important intellectual content. Arthur van Leerberghe coordinated and supervised data collection and critically reviewed the manuscript for important intellectual content. Dr Tang carried out the initial analyses and critically reviewed and revised the manuscript for important intellectual content. Dr Lu and Dr Vardi coordinated and supervised data collection and were involved in the interpretation of the data and critically reviewed and revised the manuscript for important intellectual content. Dr Schranz conceptualized and designed the study, coordinated and supervised data collection, and critically reviewed and revised the manuscript for important intellectual content. Although employees of the sponsor were involved in the design, collection, analysis, interpretation, and fact‐checking of information, the content of this manuscript, interpretation of its data, and the decision to submit the manuscript for publication were independently made by the authors. All authors approved the final manuscript as submitted and agree to be accountable for all aspects of the work.

## APPENDIX 1

**Table:** Statistical comparison of end points between the two C1‐INH doses

Efficacy end‐points	*t* test		Mixed‐effects model	
	Mean difference	*P* value^a^	Adjusted mean difference	*P* value^b^
Number of angioedema attacks	−0.40	0.035	−0.43	0.030
Cumulative attack severity	−0.66	0.055	−0.71	0.043
Cumulative daily severity	−1.85	0.045	−1.71	0.066
Number of treated acute attacks	−0.22	0.067	−0.23	0.068

C1‐INH, C1 inhibitor.

a *P* value and 90% confidence intervals are based on 2‐sided paired *t* test at *α* = 0.1.

b *P* values, 90% confidence intervals, and adjusted means are based on mixed‐effects model at *α* = 0.1, with period, treatment, and sequence as fixed effects and subject nested within sequence as random effect.
